# The macrophage: a key player in the pathophysiology of peripheral neuropathies

**DOI:** 10.1186/s12974-022-02454-6

**Published:** 2022-04-16

**Authors:** Zeina Msheik, Mohamed El Massry, Amandine Rovini, Fabrice Billet, Alexis Desmoulière

**Affiliations:** 1 UR 20218 (Neuropathies and Therapeutic Innovations), Faculties of Medicine and Pharmacy, University of Limoges, Limoges, France; 2 Department of Physiology, and UR 20218, Faculty of Pharmacy, University of Limoges, 2 rue du Docteur Marcland, 87025 Limoges Cedex, France

**Keywords:** Nerve-resident macrophages, Macrophage polarization, Peripheral neuropathy, Wallerian degeneration, Neuroinflammation, Oxidative stress

## Abstract

Macrophages are present in all mammalian tissues and coexist with various cell types in order to respond to different environmental cues. However, the role of these cells has been underestimated in the context of peripheral nerve damage. More importantly, macrophages display divergent characteristics, associated with their origin, and in response to the modulatory effects of their microenvironment. Interestingly, the advent of new techniques such as fate mapping and single-cell transcriptomics and their synergistic use has helped characterize in detail the origin and fate of tissue-resident macrophages in the peripheral nervous system (PNS). Furthermore, these techniques have allowed a better understanding of their functions from simple homeostatic supervisors to chief regulators in peripheral neuropathies. In this review, we summarize the latest knowledge about macrophage ontogeny, function and tissue identity, with a particular focus on PNS-associated cells, as well as their interaction with reactive oxygen species under physiological and pathological conditions. We then revisit the process of Wallerian degeneration, describing the events accompanying axon degeneration, Schwann cell activation and most importantly, macrophage recruitment to the site of injury. Finally, we review these processes in light of internal and external insults to peripheral nerves leading to peripheral neuropathies, the involvement of macrophages and the potential benefit of the targeting of specific macrophages for the alleviation of functional defects in the PNS.

**Graphical Abstract:**

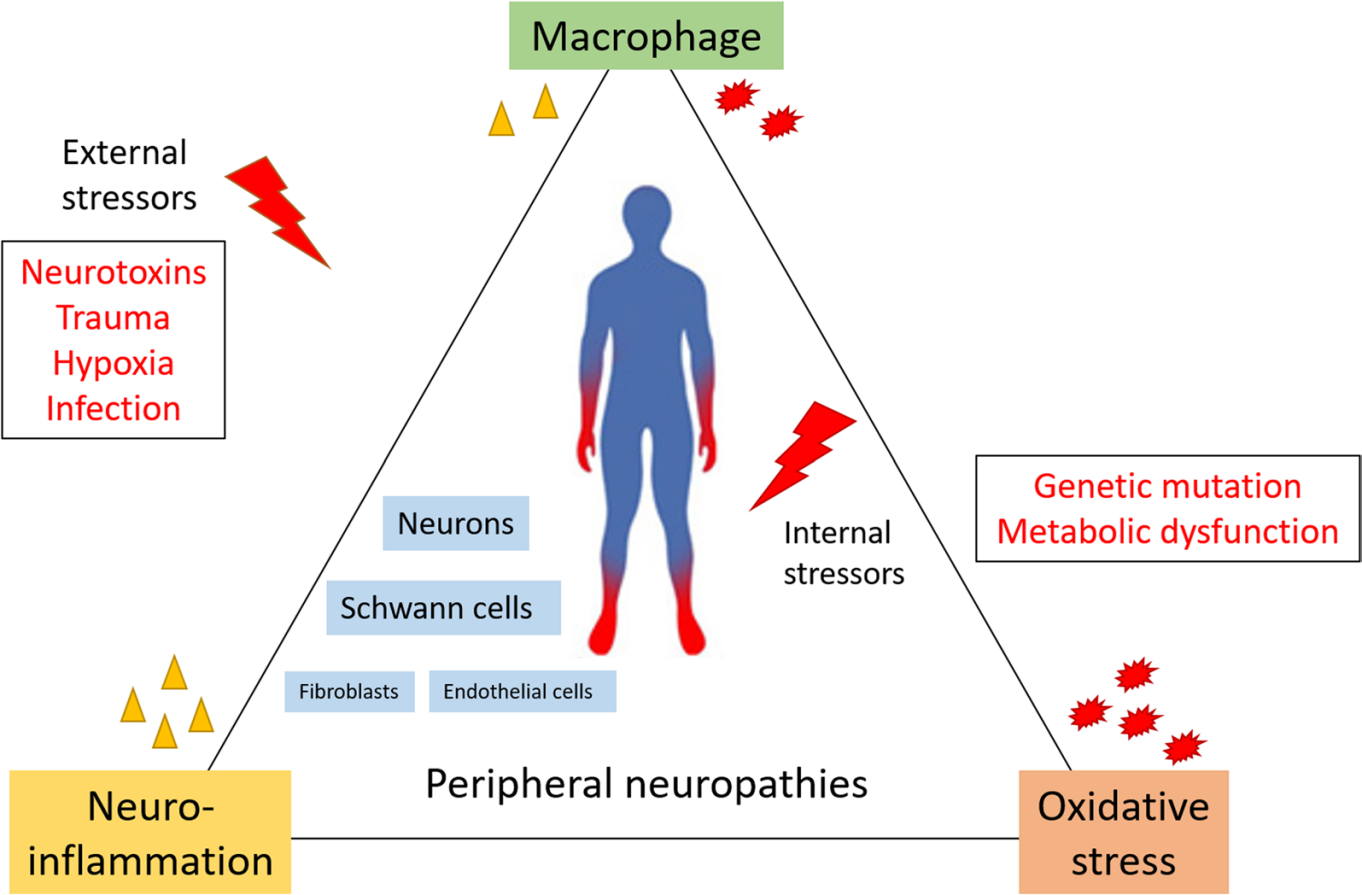

## Background

Most tissues in the body possess tissue-resident macrophage populations that are critical regulators of tissue homeostasis and host protection, in a diverse and changing environment. These cells are recognized as tissue phagocytes that derive from blood monocytes while their heterogeneity has also gained increased recognition. Despite some remaining controversies, the embryonic origin of key tissue-resident macrophage populations is now largely understood. The day-to-day maintenance and homeostasis of the resident macrophage pool is thus suggested to take place via two mechanisms: (i) proliferation of initially resident macrophages and (ii) monocyte infiltration and engraftment [1]. The question of which of these is favoured under physiological or pathological conditions in still unclear. Macrophages respond to environmental signals by reprogramming their metabolism and polarizing their phenotype/function in order to regulate their microenvironment in an active feedback loop. These cells express a plethora of cytokines, produce nerve growth factor (NGF) and ROS, and also regulate the composition of the extracellular matrix (ECM) [2–6]. In the case of injury such as trauma or infection, it is well-known that various non-neuronal cells act at the site of injury, mainly in the form of Schwann cells (SC), and immune cells such as macrophages and fibroblasts [7, 8]. Macrophages, in particular, play an orchestrating role in peripheral nervous system (PNS) tissue injury and degeneration and later in the resolution of inflammation and tissue repair. Importantly, emerging evidence suggests that the phenotype of macrophages (pro-inflammatory versus anti-inflammatory) affects the outcome of regeneration [9]. “Healthy” neuroinflammation contributes to healing and remyelination through the production of several neurotrophic factors by all types of immune cells, the reduction of immune over-activity by growth factors produced by immune cells, the phagocytic clearance of inhibitory myelin debris and toxic substances, and the removal of chondroitin sulphate proteoglycans that impede remyelination and axonal regeneration [10]. In contrast, unresolved neuroinflammation can be detrimental to nerve structure and consequently, nerve function. In models of PNS axon regeneration in rodents, the local environment is permissive to regeneration for up to 4–8 weeks after injury, and becomes less trophic or atrophic afterwards, with changes in the ECM [6]. In this regard, there is a distinction between the terms PNS “axon regeneration” and PNS “nerve repair”. Indeed, a small amount of evidence has shown that axons in the PNS may be able to regenerate and function properly after injury. However, nerve regeneration and clinical functionality after injury is probably not as optimal, hence in the best case, what occurs is rather nerve repair.

In this review, we summarize recent findings regarding nerve-resident macrophages, the current understanding of the mechanisms that underlie self-maintenance and imprinting of nerve-resident macrophages, as well as those controlling the access of monocytes to the macrophage niche. We revisit the process of Wallerian degeneration (WD), summarizing the critical steps and thereby the accompanying events of monocyte/macrophage recruitment into injured nerves, while taking into account the importance of the nerve–blood barrier in this process. Finally, we discuss the main PNS pathologies, specifically addressing the role of neuroinflammation and oxidative stress in their evolution and progression. Although many issues remain to be adequately resolved, new techniques are increasing our understanding of the role of macrophages in immunity and immunopathology, and additionally providing detailed insights on macrophage commitment to tissue niches and their behaviour in the case of internal or external insults. This, it is hoped, will pave the way for targeting of specific macrophages for alleviating nerve injuries associated with peripheral neuropathies.

## Tissue macrophages: ontogeny and function

### What we know about their origin

In 1926, Alexis Carrel and Albert H. Ebeling concluded after a series of experiments that: “a macrophage is merely a monocyte in a more active metabolic condition” [11]. Since then, the prevailing view has been that tissue macrophages are continuously repopulated by blood-circulating monocytes derived from progenitors present in the bone marrow. In 1972, a group of specialists from around the world proposed the mononuclear phagocyte system (MPS) as a classification of both monocytes and macrophages and their precursors. These authors described a family of cells based on their origin, morphology, function and kinetics [12]. Indeed, monocytes and macrophages rely on the same growth factors and transcriptional regulators (such as Purine-rich Box-1 (PU.1)), and share the expression of several surface markers (notably colony stimulating factor-1 (CSF1) receptor) [13]. However, in the last decade, this view has been revised after the emergence of a series of breakthrough publications. Impressive evidence, based on macrophage ontology, showed that some tissue-resident macrophages are first seeded during embryonic haematopoiesis without monocyte intermediates, and that these persist throughout the individual’s life. As such, they are able to locally self-maintain independent from blood monocyte input [14–18]. Given that haematopoiesis is well-conserved between Drosophila and vertebrates, studies in Drosophila larva showed that primitive haematopoiesis in the yolk sac produces erythroid cells and macrophage progenitors as the sole “white blood cells”. These primitive macrophages give rise to many types of tissue-resident macrophages [19]. More recently, several fate mapping ontogeny studies have been performed in embryonic and adult mice [20–22]. Foetal macrophage ontogeny showed that three successive waves of haematopoiesis (from embryonic day 7.5 (E7.5) till E10.5) occur during development (reviewed in [15]). The first wave is termed primitive haematopoiesis, taking place in the extra-embryonic yolk sac and giving rise to maturing macrophages. The second wave is intraembryonic and arises from erythro-myeloid progenitors (EMP) that migrate to the foetal liver and give rise to maturing monocyte/macrophages. The third wave arises from haemogenic endothelium and leads to the generation of immature haematopoietic stem cells (HSCs) that colonize the foetal liver as well as the foetal bone marrow. Eventually, mature HSCs in the bone marrow commit to their role in adult haematopoiesis. Importantly, many adult tissue-resident macrophages have been traced to embryonic origin, independently of provision from the bone marrow [15, 20].

The contribution of monocyte recruitment and resident macrophage proliferation to expansion of tissue macrophages is still controversial. To date, available evidence suggests that these two mechanisms are unlikely to be independent in the course of local macrophage expansion (for review, see [23]). In general, it is accepted that, at steady state, (1) embryonic macrophages, (2) adult monocyte-derived macrophages, and (3) daughter cells of adult monocyte-derived macrophages function all function together. In addition, in inflamed tissue, (4) recruited adult monocyte-derived cells are added to the already-diverse population [15]. However, for a given tissue, the contribution of distinct macrophage lineages/subtypes in these processes is currently unclear. To address this question, sophisticated approaches (such as fate mapping) are being developed and used to separate resident from infiltrating macrophages [23]. In their review, Ginhoux and Guilliams [15] classify adult tissues into those which exclusively contain yolk-sac-derived macrophages (brain, lung, liver, and epidermis), those with fast steady-state monocyte recruitment (dermis and gut), and tissues with slow steady-state input (pancreas and heart). Steady, low-grade metabolic, oxidative, or mechanical stress (as in physiological aging) can deplete these tissue-macrophage pools. Therefore, these conditions could drive homeostatic monocyte provision in order to replenish tissue-macrophage pools in a pattern similar to inflammation.

### Macrophage function: far from being dichotomous

Macrophage polarization is the term used to describe the remarkable plasticity of macrophages and their phenotype switch in response to microenvironmental cues [24]. In case of injury, recruited monocytes and tissue-resident macrophages proliferate and undergo marked alterations in cell surface markers and function, then regulating inflammation and ultimately tissue repair or fibrosis [25]. Based on in vitro induction experiments, activated macrophages can be generally divided into two subtypes: M1-like macrophages and M2-like macrophages, with the difference between the two cell populations summarized in Table 1 [26]. The M1/M2 nomenclature was established analogous to the Th1/Th2 dichotomy. Classical macrophage activation is characterized by high antigen-presenting capacity and activation of polarized type 1 response (hence M1) [27]. M1 macrophages express pro-inflammatory markers such as Nos2, Arg1, Ccl2, Ccl7, Il1α, and Alox15 [28]. They also show increased production of reactive oxygen and nitrogen species (ROS and RNS, respectively) upon stimulation by pathogen- or damage-associated molecular patterns (PAMPs or DAMPs, respectively) [24, 27, 29]. On the other hand, alternatively activated/deactivated macrophages which adopt an anti-inflammatory phenotype are termed the M2 family. M2 macrophages are divided into: M2a (where ‘a’ stands for alternative), induced by interleukin (IL)-4 or IL-13; M2b, induced by exposure to immune complexes and agonists of Toll-like receptors (TLRs) or IL-1R; M2c, induced by IL-10 and glucocorticoid hormones [27]; and M2d, induced by TLR antagonists to secrete IL-10 and vascular endothelial growth factor (VEGF) [30]. The nuclear transcription factor PPARγ controls the direction of macrophage polarization by promoting the M2 cell phenotype and suppressing the switch to an M1 phenotype. In contrast, stimulation of the NF-κB signalling pathway promotes the polarization to M1 macrophages, while its inhibition promotes the M2 phenotype [26].

**Table 1:**
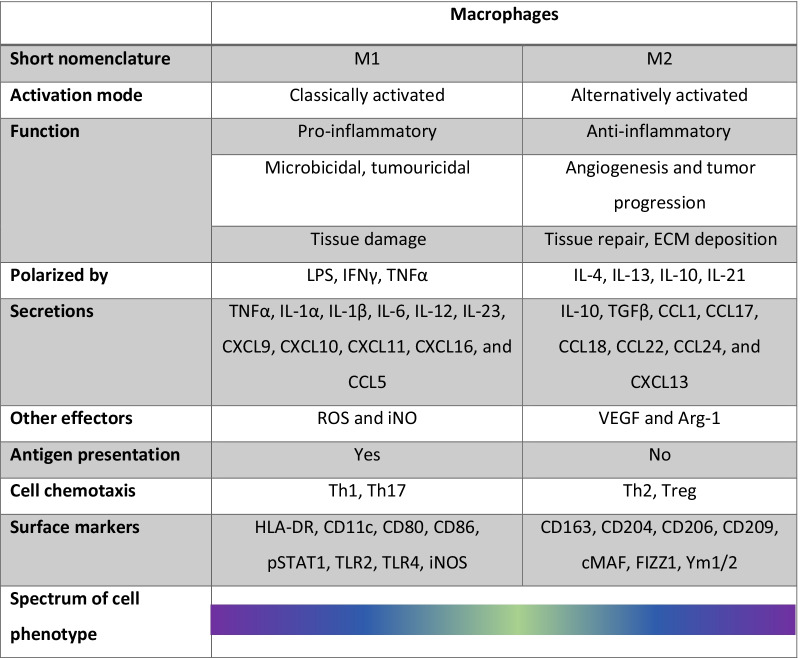
Comparison between features of classically activated (M1) and alternatively activated (M2) macrophages

*ECM* extracellular matrix, *LPS* lipopolysaccharide, *IFN* interferon, *TNF* tumour necrosis factor, *IL* interleukin, *CXCL* chemokine (C-X-C motif) ligand, *CCL* C–C motif chemokine ligand, *TGF* transforming growth factor; *ROS* reactive oxygen species, *iNO* inducible nitric oxide, *VEGF* vascular endothelial growth factor, *Arg-1* arginase-1, *Treg* regulatory T lymphocyte, *pSTAT* phosphorylated signal transducer and activator of transcription, *TLR* toll-like receptor, *iNOS* inducible nitric oxide synthase, *FIZZ1* found in inflammatory zone 1

To date, fully polarized “mature” M1 and M2 cells in their various versions seem to be the extreme ends of a continuum; and macrophage populations are far from being stable subsets, but cells which actively modulate their phenotypes in response to tight physical, chemical, and cell-to-cell cues [31, 32]. In addition, many studies find the in vitro M1/M2 classification problematic when it comes to applying it in vivo [31, 33, 34]. The use of adapted and synergistic techniques, such as single-cell transcriptomics, proteomics, fate mapping, and imaging, is thus increasingly important for investigating complex macrophage populations [31, 35]. Moreover, the range of markers used to identify macrophages, in vivo/in vitro differences, as well as interspecies variability, should be kept in mind when analysing the results. Finally, disorders or shifts in macrophage polarization may be correlated with the pathogenesis of particular diseases, such as cancer. In this regard, modulating macrophage polarization by cytokines, chemical compounds, and nano-carriers is currently under investigation as a potential treatment strategy [26].

### Macrophage tissue identity and niche

Macrophages are considered to be the most plastic cells of the haematopoietic system [25]. Essentially, tissue macrophages are not all alike as they adopt tissue-committed roles, e.g. Kupffer cells in the liver, microglia in the brain, and nerve-associated macrophages in the PNS. The particularity of the PNS is that peripheral nerves are spatially distributed throughout the whole body and innervate various tissue types. A description of nerve-associated macrophages in different tissues is reviewed in [36]. Whether these macrophages have a PNS-specific identity or rather host tissue-signature is a matter of debate, although both organ- and nerve-specific cues are likely to influence resident macrophage identity. In adult tissue, multiple populations of macrophage-like cells coexist and perform critically timed functions [14, 17, 35, 37]. Strictly linking the identity of tissue-resident macrophages to an embryonic origin is still a hotly debated subject [38]. In addition to ontogeny, it is well-known that microenvironmental cues, including trophic factors, ECM scaffolds, stromal cells, and the vasculature, tightly regulate the development and homeostasis of all cell types. Therefore, the same is true of macrophages in that the tissue “niches” in which they reside modulate their function [38]. These tissue-specific processes are governed by mechanisms such as DNA methylation, histone modification and chromatin structure (reviewed in [39]). For instance, 12,743 enhancers (genomic transcriptional regulatory elements) were identified as being macrophage-specific in comparison to monocytes and neutrophils in mice. Of these, less than 2% were found to be common across all tissue-macrophage populations (represented by microglia, Kupffer cells, spleen, lung, peritoneal, ileal and colonic macrophages) [40]. Moreover, when fully differentiated macrophages were transferred to a different tissue, their expression profile was reprogrammed [17, 40]. Therefore, epigenetic regulation regulated by both tissue- and lineage-specific transcription factors shape macrophage tissue identity.

In particular, CSF1 (also known as macrophage colony stimulating factor (M-CSF)) is critical for the development, maintenance, and density control of macrophages in most tissues. CSF1 exists in three forms: a secreted glycoprotein, a secreted chondroitin sulphate proteoglycan, and a cell surface glycoprotein [41]. Several studies in rodent models have assessed the role of each of the three isoforms of CSF1 in macrophage homeostasis in different tissues. The conclusion was that at steady state, a local maintenance equilibrium exists between macrophages and CSF1, and controls the levels of the latter. When local macrophage number decreases (as in physiological cell death), local CSF1 concentrations increase triggering the proliferation of existing macrophages. Daughter macrophages then start to consume CSF1, eventually restoring the equilibrium [1]. On the other hand, according to the territory model that has been recently proposed [42], the optimal pattern of spatial distribution of macrophages is governed by mutual repulsion, i.e. by contact inhibition. Using highly motile filopodia, each macrophage actively surveys its milieu to define its territory [43], which, depending on the tissue type, can vary in area [42]. The authors speculated that this concept could explain monocyte infiltration during inflammation, which is upon the loss of resident macrophages in a particular territory [42]. Recently, Guilliams and colleagues [1] emphasize the nurturing and tuning role of stromal cells for immune cell maintenance and function. They summarized that: the haptotactic niche is created mainly by (1) local fibroblasts that secrete CSF1 and (2) ECM with bound CSF1, which regulates macrophage arrangement, without ignoring the role of contact inhibition in controlling cellular density in the tissue niche [1]. Indeed, as cell survival and function cannot be separated from their context, i.e. the microenvironment, the nurturing niche theory fits well the current knowledge and understanding of macrophage biology and heterogeneity.

For decades, CSF1 and its receptor (CSF1 receptor (CSF1R)) were considered to be the chief regulators of cell differentiation in the MPS. CSF1R was identified as being also activated by a novel cytokine: IL-34 [44, 45]. The existence of two ligands, with distinct amino acid sequences, for CSF1R raises several questions. Several studies have indicated that IL-34 can bind to CSF1R with higher affinity and induce stronger tyrosine phosphorylation of CSF1R and downstream molecules than its counterpart CSF1 [44, 46, 47]. Through alternative mRNA splicing, two secreted isoforms of IL-34 are generated [48]. Recently, Ogawa and colleagues [49] suggested the existence of a third isoform bound to the plasma membrane of follicular dendritic cells. The molecular mechanisms that control IL-34 expression, physiologically or pathologically, remain unclear. Importantly, IL-34 has been shown to support the survival of human monocytes and promote the formation of macrophage colonies in human bone marrow cell cultures [47]. Interestingly, identical effects of IL-34 and CSF1 were shown in human monocyte signalling and differentiation levels [47, 50]. Nevertheless, CSF1 and IL-34 show differences in effects on macrophage polarization. Monocytes differentiated into macrophages by either CSF1 or IL-34, and then polarized into M1 or M2 phenotype using LPS/IFNγ or IL-4 show a distinct cytokine secretome [50]. This suggests that macrophages generated in both conditions may have different roles in pro- or anti-inflammatory pathophysiological conditions. Whereas IL-34 transcripts can be detected in many tissues throughout the body, IL-34 protein can be recognized in a tissue-specific manner; notably in keratinocytes of the skin and neurons of the brain [51]. Investigating the potential role of IL-34 in controlling macrophage “niche”, in analogy to the theories proposed for CSF1, would be complementary, especially in the PNS, where macrophage polarization appears to be affected by multiple factors.

### Monocytes, macrophages, and reactive species

ROS and RNS secretion is important in monocyte/macrophage surveillance in tissue homeostasis and first-line defence. Under physiological conditions, around 90% of endogenous ROS are produced in mitochondria by oxidative phosphorylation [52], and a minor amount comes from plasma membrane proteins, lipid metabolism, and the activity of cytosolic enzymes [53]. Normally, the body is equipped with a counter-acting defence system (such as glutathione and cytochrome p450) to “balance” continuous physiological ROS production. Nevertheless, the balance between ROS generation and the antioxidant system is slightly in favour of ROS, and a continuous low level of oxidative damage persists [54]. In turn, activated circulating monocytes and M1 macrophages increase ROS and RNS production after exposure to various signals, including pathogen-derived molecular patterns (PAMPs, such as lipopolysaccharide), damage-associated molecular patterns (DAMPs, such as high-mobility box 1 protein (HMGB-1), nucleotides, and DNA), cytokines (e.g. tumour necrosis factor-α (TNFα), interferon-γ), metabolic stress (e.g. hyperglycaemia, advanced glycation end-products, oxidized lipoproteins), endoplasmic reticulum stress, unfolded/misfolded protein accumulation, and some nanoparticles [29].

While both monocytes and macrophages are also subjected to oxidative damage caused by their own secretion of reactive species, macrophages are more robust than monocytes [55]. In order to survive and continue to function in such a hostile oxidative and inflammatory environment, macrophages are equipped with a network of protective mechanisms (notably the Nrf2 pathway and its downstream effectors, and the FOXO pathway) [29]. This difference may be linked to the modulatory effect of macrophages in which they kill excess monocytes during an inflammatory response. However, it is noteworthy that the mechanism of ROS sensing by macrophages (and other cells) is not fully clear. Despite this advantage in being protected from oxidative damage, macrophages are still not fully resistant to ROS-induced death. When oxidative stress is maintained for a long period of time, macrophages accumulate massive amounts of oxidized proteins and lipids, leading to metabolic dysfunction, phenotypic alterations and cell death. This is particularly relevant to human diseases such as inflammatory, autoimmune, cancerous, vascular, respiratory, and neurodegenerative diseases in which ROS are produced excessively [53]. Moreover, over time, innate repair mechanisms tend to be slowed and then overwhelmed, and organisms progressively lose physiological integrity due to the accumulation of cellular damage, contributing to aging and age-related diseases such as neurodegeneration.

## Nerve-associated macrophages: where we are now

It is now four decades since sciatic nerve macrophages were first identified [56]. However, and in compelling contrast to central nervous system (CNS) microglia, PNS-resident macrophages are among the least studied subpopulations. Little is known about their cellular origin, capacity for self-maintenance, and their gene signature. Most research efforts are devoted to study CNS pathological mechanisms, where macrophages play pivotal roles. Recent observations in many CNS neurodegenerative diseases that PNS is a relevant disease target-especially in Multiple Sclerosis [57] could reshape research towards investigating systemic involvement in neurological diseases.

Where are these macrophages located? A peripheral nerve is made up of several bundles of axons, where individual axons are enveloped by myelinating or non-myelinating SC. Axons are embedded in an endoneural compartment made up of collagenous connective tissue and fibroblasts [58]. Axon fascicles are enclosed in the collagenous epineurium matrix that also contains small arteries and veins, as well as fibroblasts and macrophages [59–61]. Studies also show that macrophages exist in the endoneurium, between myelin sheaths and in close contact with axons [56, 59, 62]. Normally, these macrophages continually survey the environment for threats and help to clear cellular debris.

Single-cell RNA sequencing, first used in 2009 [63], allows the remarkably fine characterization of specific cell subsets in a heterogeneous tissue. Only recently, this technique was used to characterize nerve-associated macrophages in mouse skin [35]. These cells are distinguished by their high expression of CX3CR1 marker (CX3CR1^hi^), in comparison to other dermal macrophages. CX3CR1^hi^ nerve-associated macrophages closely interact with sensory nerves, both with respect to their transcriptome and their axon-scanning behaviour. Using fate mapping techniques, these cells were found to arise either from CX3CR1^hi^ prenatal progenitors, which are the dominant source in homeostasis, or from CX3CR1^low^ pro-inflammatory monocyte-derived macrophages [64] in the case of tissue injury; thus two distinct origins of these nerve macrophages can be differentiated. In an ear-punch murine model, these cells expand and contribute to myelin degradation, nerve regeneration, and wound healing [35]. The role of CX3CR1 in these highly committed peripheral nerve macrophages is not yet fully clear. Evidence from murine models indicates that CX3CR1 ligand CX3CL1 (also known as fractalkine FKN) triggers ROS production in CX3CR1 macrophages, as well as monocyte infiltration in sciatic nerves [65]**.** In addition, the chemokine FKN was shown to mediate neural/microglial interaction in neuropathic pain in rats [66].

**Fig. 1:**
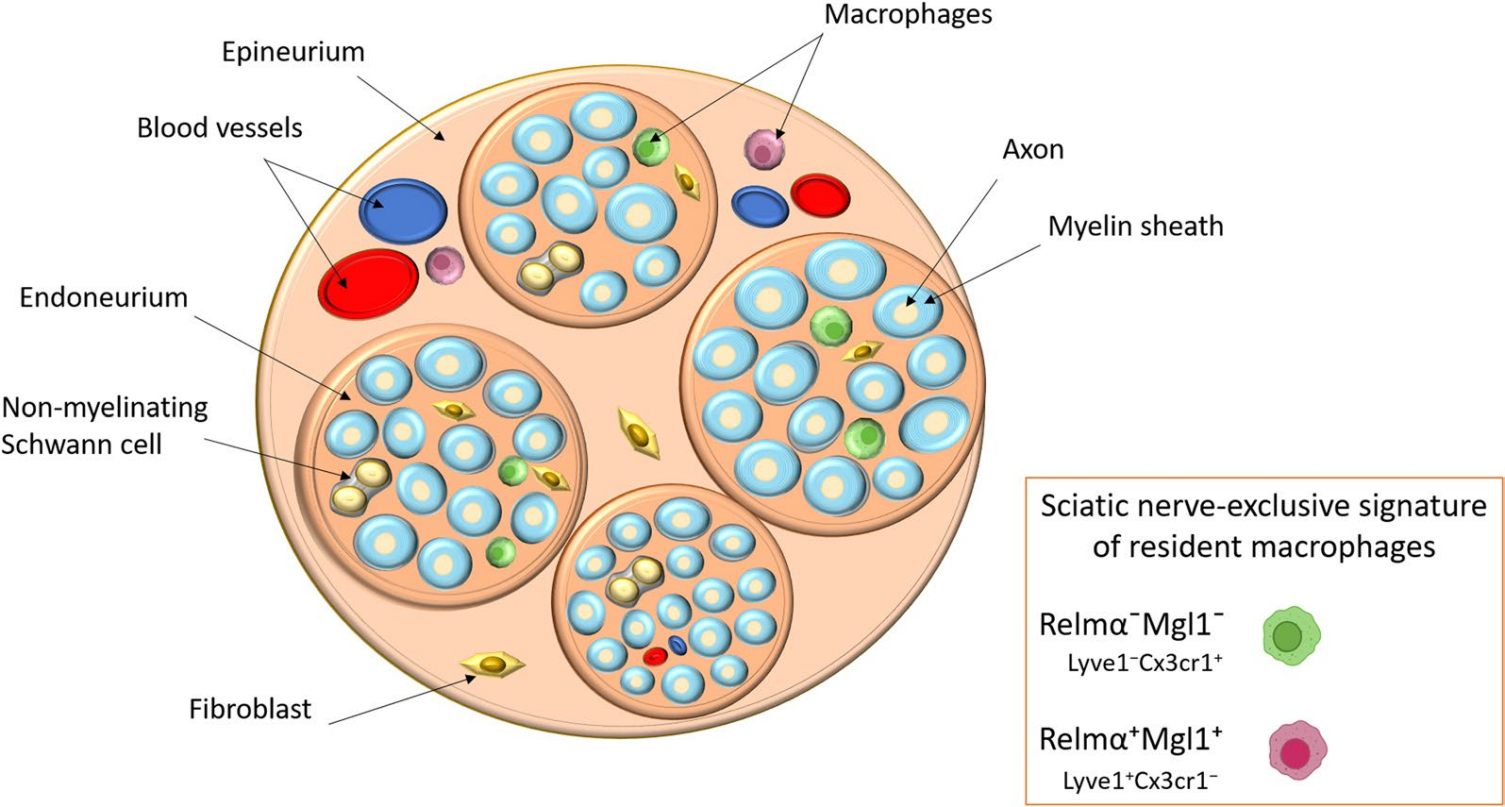
Schematic cross section of the sciatic nerve**.** RelmαMgl1 is an exclusive “fingerprint” of resident macrophages in the sciatic nerve in normal physiological state. Using confocal microscopy, Relmα^−^Mgl1^−^ Lyve1^−^Cx3cr1^+^ macrophages are located inside the endoneurium and are more abundant than Relmα^+^Mgl1^+^Lyve1^+^Cx3cr1^−^ macrophages found in the epineurium connective tissue. The epineurial Relmα^+^Mgl1^+^ sciatic nerve macrophages were often found to be associated with blood vessels, a characteristic not observed in endoneurial Relmα^−^Mgl1^−^ macrophages [34]. The scale is arbitrary for visual clarity reasons

A recent study thoroughly characterized sciatic nerve macrophages in mice [34]. Sciatic nerve macrophages presented a tissue-specific gene signature distinguishing them from other tissue macrophages (in the optic nerve, liver, lung, spleen, and peritoneum) and CNS microglia. The sciatic nerve macrophage profile was enriched by a unique gene set including *Adam19*, *Cbr2, Cd209d, Foxred2, Fxyd2, Mgl2, Mmp9, Il1rl1, Kmo* and *Tslp.* Single-cell profiling identified two spatially separated sciatic nerve macrophage types: Relmα^+^Mgl1^+^ cells in the epineurium and Relmα^−^Mgl1^−^ cells in the endoneurium (Fig. 1). By fate mapping, the authors showed that sciatic nerve macrophages do not derive from the early embryonic precursors colonizing the CNS, but originate primarily from late embryonic precursors and are replaced by bone marrow-derived macrophages over time. Finally, in contrast to the CNS, upon injury, the PNS macrophage pool is replenished by monocyte-derived macrophages. Indeed, investigating nerve-associated macrophages is a very topical area of research with significant challenges. Recently published studies, although few, have contributed greatly to the understanding of the striking functional plasticity of macrophages and of their potential role in peripheral neuropathies. Studying peripheral nerve immune responses in conditional knockout transgenic animals has provided further strong insights to aid the understanding of physiological/pathological crosstalk between the systemic immune system and peripheral nerve compartment.

## Peripheral nerve disease: all roads lead to inflammation

### Wallerian degeneration (WD): implications of neuronal immune response and macrophages

**Fig. 2:**
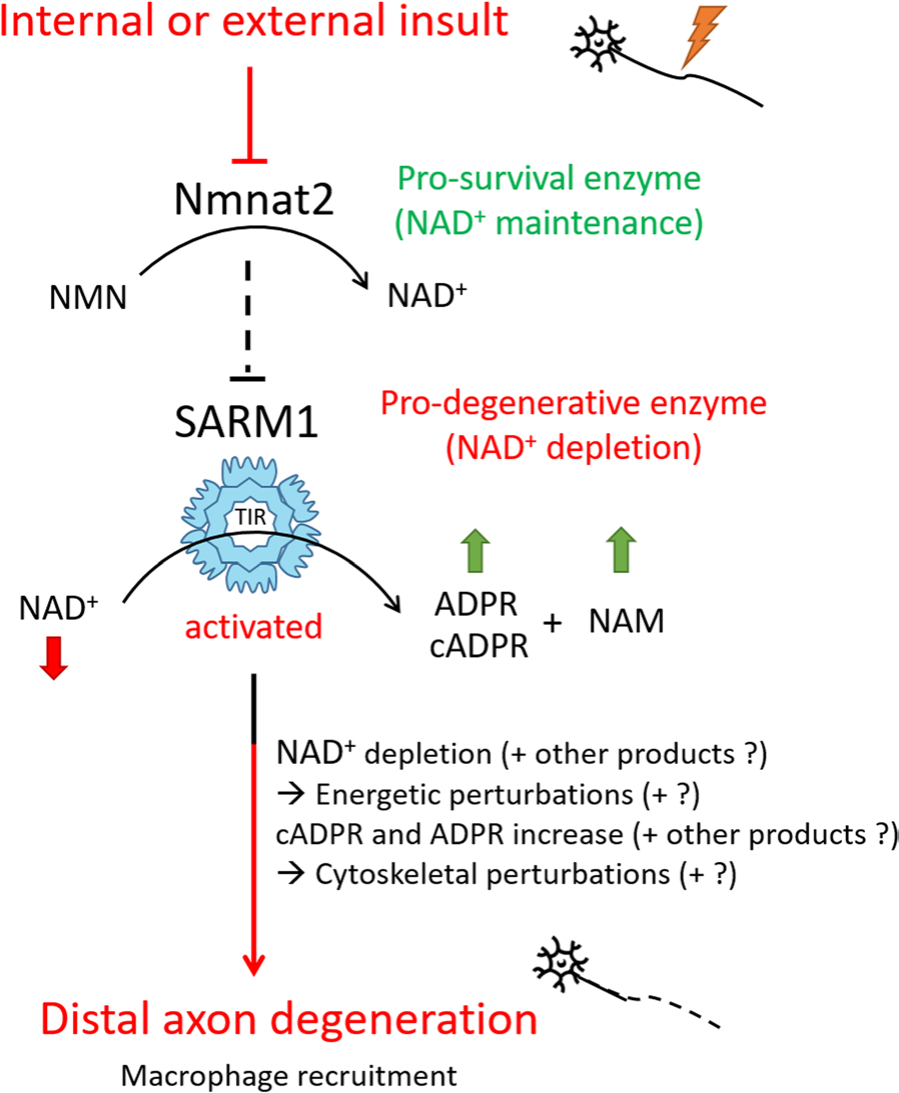
A schematic representation of the key players along the Wallerian degeneration pathway. Nmnat2 activity can be disrupted by intrinsic causes (*Nmnat2* gene mutation, Nmnat2 inhibition, or axonal failure) or by extrinsic insults due to axonal injury. Nmnat2 is the normal axonal synthetic enzyme for NAD^+^ (nicotinamide adenine dinucleotide). When Nmnat2 activity is stopped, SARM1 is activated and through dimerization of its Toll/interleukin-1 receptor (TIR) domain, triggers a rapid breakdown of NAD^+^ into ADPR (adenosine diphosphate ribose), cADPR (cyclic adenosine diphosphate ribose), and nicotinamide (NAM). Exactly how SARM1 is activated is still debated. One explanation is that the accumulation of Nmnat2 substrate, nicotinamide mononucleotide (NMN), can activate SARM1 [85]. NAD^+^ depletion (through both blockade of Nmnat2 and activation of SARM1) and ADPR /cADPR accumulation (products of SARM1) are suggested to cause energetic and cytoskeletal perturbations, respectively, thereby mediating axon degeneration distal to the injury site. This is followed by SC activation and macrophage recruitment

This process, initially named for Augustus Volney Waller who experimented on and described distal axon and myelin disintegration following nerve transection in 1850 [67] is now recognized to occur in other non-injury neuropathies, such as Alzheimer’s disease [68]. Immediately after injury, and before transcriptional changes take place in the injured neuron, a retrograde injury signal is sent towards the nucleus [69]. The length of human axons presents a special challenge in comparison to animal axons, as injury signals must travel a long distance to reach the nucleus, located in the brain or spinal cord. Subsequently, the neuron upregulates the expression of growth-associated genes [9]. Meanwhile, the distal fibre ending undergoes “dying-back” degradation and clearance. The unusual extended survival of the *slow Wallerian degeneration* (Wld^S^) mutant mouse axons without their cell bodies [70] has fundamentally changed our view of axon degeneration. Subsequent studies have indicated that the Wld^S^ gene product is an extraordinary fusion product of the ubiquitin ligase Ube4b gene and Nmnat1 (nicotinamide mononucleotide adenylyltransferase 1) [71]. This has resulted in a deeper understanding of WD at the molecular level. Specifically, maintaining axonal NAD^+^ levels by Nmnat2 enzyme activity is essential for axon protection [72]. Since that time, Sarm1 ((sterile α/Armadillo/TIR homology domain) was identified as a pro-degenerative axonal factor preserved throughout evolution [73]. A new piece has also been added to the puzzle with the discovery of the NAD^+^-depleting activity of SARM1 [74] acting downstream from Nmnat2 [75]. Figure 2 outlines the main players that control the WD pathway.

The recent discoveries of a link between *Nmnat2* mutation and human diseases, in addition to the determination of protein structures involved in WD, is indeed promising in terms of deciphering underlying pathological mechanisms as well as for aiding drug development [68]. On the other hand, it has been well-established that the absence of Sarm1 in Drosophila [73], mouse [76] and Zebrafish [77] prevents the degradation of damaged axons. In addition, reducing or blocking the expression of Sarm1 ameliorates SC-resistance to chemotherapeutic agents, even after axon injury. However, macrophage recruitment to the injury site, and thus resolution of focal damage, was not affected [77]. Indeed, our knowledge to date is far from complete, and it is accepted that this process appears to be driven by a complex combination of cell populations to perform a wide spectrum of tasks at the site of injury. For instance, WD of the axon is accompanied by the trans-differentiation of SCs into a proliferative and repair phenotype, and the activation of a sterile immune response, specifically macrophages that are recruited by mechanisms that are both dependent and independent of blood monocyte infiltration [8, 14].

What attracts macrophages to the site of injury? After nerve insult, macrophages are among the first (2–3 days post-injury) and the most abundant cells to infiltrate the injury site, where monocytes/macrophages are recruited by factors produced by repair SCs, and then they further produce chemoattractants, such as CCL2, TNF-α, IL-1α and IL-1β, for greater macrophage infiltration [78]. CCL2 is the major chemokine for monocytes and macrophages. It binds with high affinity to the absence of receptor CCR2 which is expressed essentially by monocytes and macrophages [79] as well as by sensory neurons after injury [80]. Wang and colleagues [81] examined the immune capacity of neurons, particularly the role of SARM1 in Ccl2 induction. They reported that SARM1 acts via c-Jun kinase (JNK) and phospho-Jun to trigger the expression of CCL2 as well as CSF-1, CCL7, and CCL12 in DRG neurons. This neuronal immune response starts as early as 8 h post-injury, that is, even before the axon starts to degenerate. Moreover, blockade of the SARM1-JNK pathway abolishes the recruitment of immune cells as well as axon degeneration. However, genetic deletion of c-Jun has no effect on axon degeneration [82], and c-Jun deficiency effectively suppresses the neuronal immune response [81]. Thus, c-Jun directly targets the genes of immune factors expressed by the injury-afflicted neurons, only under pathological conditions.

On the other hand, it is worth mentioning that macrophages also express SARM1, which initially regulates the recruitment of transcription factors and RNA polymerase II to the Ccl5 promoter. It has been previously shown that Sarm1^−/−^ bone marrow-derived macrophages exhibits reduced Ccl5 expression compared with WT cells, for both TLR-dependent and -independent stimulation of cells [83]. However, more recently, Doran and colleagues [84] used CRISPR/Cas9-mediated SARM1 knockout and epitope-tagged mice to show that SARM1 is indeed expressed in macrophages but does not regulate nuclear transcription.

### Blood–nerve barrier and neuroinflammation

Normal axonal conduction requires good regulation of the endoneurial microenvironment, i.e. cells or cell parts (axon, myelin sheath, fibroblasts, and macrophages), connective tissue, and solutes. Furthermore, this area of high metabolic demand implies an active functional and anatomical relationship with the vascular compartment [86]. Endoneurial homeostasis is achieved by endoneurial microvascular endothelia that form tight junctions controlling ion, solute, water, nutrient, macromolecule and leukocyte movement between the bloodstream and the endoneurium. This blood-nerve barrier (BNB) is considered to be the second most restrictive vascular system after the blood–brain barrier (BBB), based on classic in situ permeability studies [60]. Passage across the BNB can be attributed to relatively lower levels of P-glycoprotein transporter activity in comparison to the BBB, thus limiting the efficiency of xenobiotic and neurotoxin removal [87]. Furthermore, in several human peripheral neuropathies, structural alterations in the endoneurial microvessels or interactions with haematogenous immune cells have been described. Neuroinflammation, which is currently recognized as a hallmark of virtually all neurological disorders [10], increases vascular permeability and disrupts the BNB [88]. In addition, oxidative stress induces a downregulation of the tight and adherens junction proteins [89]. These processes consequently allow neurotoxins, endotoxins and inflammatory cells to invade. However, our knowledge about mechanisms of haematogenous leukocyte trafficking at the human BNB is remains inadequate due to phenotypic and functional differences between endothelial cells from different tissues, as well as between different species [60]. Nevertheless, macrophages appear to closely interact with the vascular compartments in order to drive the inflammatory process. Indeed, macrophages at the injury site selectively sense local hypoxia and secrete VEGF to polarize the neighbouring vasculature [78]. Additionally, all infiltrating monocytes essentially express VEGF to further guide vascular sprouting. Blood vessels direct the migrating cords of SCs that are necessary for guidance of regrowing axons [34]. In addition, ECM proteins, such as collagen VI, modulate cellular changes at the injury site. Collagen VI, produced by both repair phenotype SCs and local macrophages, acts not only as a chemoattractant for monocytes/macrophages, but also modulates the secretion of other factors. Known for their potent phagocytic action, macrophages are crucial for clearing inhibitory myelin debris, and thus allowing regeneration [8, 9]. Macrophages interact with SCs to modulate their function. All of these cellular changes and interactions are mediated by the secretion and production of various cytokines, chemokines, regeneration factors, ROS, and ECM molecules [8], which all form the “soup” of degeneration/regeneration adaptive processes.

Alterations in vascular permeability in the context of peripheral nerve diseases is not fully clear. In fact, on one hand, increased permeability of the BNB implies an augmented monocyte infiltration, hence an exacerbated immune reaction. On the other hand, macrophage secretions could act as polarizing agents that assist in the regenerative process following nerve injury. Indeed, their contribution to nerve disorders is gaining more interest and targeted therapies are under consideration for macrophage-associated pathologies including peripheral neuropathies.

### Neuroinflammation and oxidative stress

Neurodegeneration is almost always accompanied by oxidative stress. In general, oxidative stress is well-recognized as a contributing factor to neurodegeneration [90]. Dying cells and degrading axons produce high levels of ROS, which are then released into the extracellular compartment. Excessive ROS production is deleterious for cells, and since myelin is rich in lipids, SCs are particularly susceptible to lipoperoxidation. External and internal stressors, such as inflammation, disrupt the balance between pro and anti-oxidant systems leading to an oxidative burst [91]. ROS were found to be elevated in sciatic nerves of CMT1A rats [92] as well as in sciatic nerves after crush injury [93] and of diabetic mice [94]. Moreover, sciatic nerves exposed to stress by non-freezing cold presented high ROS production associated with reperfusion injury and pathological destruction [95]. Hervera and colleagues [96] showed that ROS production in the injured sciatic nerve and DRG requires CX3CR1-dependent recruitment of inflammatory cells. Injury-induced ROS, as well as SC-secreted chemokines, then leads to the recruitment of inflammatory cells, including monocytes [97] and macrophages [77]. ROS alters macrophage differentiation by interfering with epigenetic (re)programming, and favouring the induction of pro-inflammatory M1 macrophages [98]. In turn, macrophages secrete ROS due to the stimulation of endothelial-derived FKN. ROS then activate transient receptor potential ankyrin 1 (TRPA1) channels on sensory neurons, which evokes a pain response [99]. It is well-known that neuronal oxidative stress activates the ERK signalling pathway that is detrimental to neurons and promotes their cell death [100]. These data, along with data collected from different organ systems, suggest that oxidative stress and neuroinflammation could potentiate each other. More importantly, the macrophages appear to play an essential role in the mediation of nerve response to injury, potentially aggravating the progression of peripheral nerve diseases.

## Peripheral neuropathies

The role of CNS microglia in neuropathic pain was first established in 2003 after observing the striking increase in microglial expression of purinoreceptor P2X_4_R after spinal cord injury in rats [101]. Later, more evidence appeared supporting immune cells not being passive observers in the nervous system, but rather active influencers in the initiation and/or progression of neuropathies. Specifically, macrophage activation contributes to experimental neuropathic pain states by releasing potent pro-inflammatory mediators, including TNFα, IL-1β, MCP-1, NGF, nitric oxide (NO) and prostanoids [102]. Moreover, peripheral nerve injury induces an increase in CSF1 in the DRG and spinal cord which is associated with induction of neuropathic pain [103].

The sciatic nerve (*Nervus ischiadicus*) crush is one of the most common models of peripheral nerve injury in rodents. It is a valuable model to study peripheral axon regeneration, as well as providing insights into the failure of CNS axon regeneration [104]. More importantly, this model has served as a valuable tool to study the involvement of macrophages in the inflammatory and regenerative responses following nerve trauma. After sciatic nerve axonotmesis in mice, pro-inflammatory mediators such as IL-1β, Cox2, and TNF-alpha are rapidly produced (within 5–10 h) [5], as well as chemokines such as MIP-1α and monocyte chemoattractant protein 1 (MCP-1) (peaking at 24 h) [4]. Furthermore, after acute peripheral nerve injury, the expression of pro-inflammatory cytokines was seen to return to basal levels two days post-injury. Interestingly, Ydens and colleagues [105], showed that M2-like macrophages were triggered, showing a strong up-regulation of tissue repair markers (arginase-1, Ym1, and Trem2), thereby limiting the attack of the pro-inflammatory surge, resulting in a neuroprotective environment. The main cells acting at the site of injury were found to be resident endoneurial macrophages (9.5-fold above baseline). Their response was rapid: 2–3 days post-crush, while haematogenous macrophages started to appear at day 4 [106]. To evaluate the effect of injury on the recently characterized sciatic nerve macrophages, Relmα^+^Mgl1^+^ and Relmα^−^Mgl1^−^ (see above, and Fig. 1), a sciatic nerve crush mouse model was established. In the distal part of the sciatic nerve, Relmα^+^Mgl1^+^ cells did not respond, i.e. did not change their gene expression profile; whereas Relmα^−^Mgl1^−^ cells rapidly responded [34]. Interestingly, this could be expected as Relmα^−^Mgl1^−^ endoneurial cells were found to be in closer contact with the injured axon and transdifferentiating repair SCs. These cells then phagocytose debris at the injury site, and contribute to the disintegration of distal axons. While MHC class II expression is correlated with classical M1 phenotype, high or low MHC class II expression did not correlate with altered macrophage activation state at different time points after sciatic nerve crush. In addition, in this study, Relmα, Mgl2, Ym1 and arginase that are considered prototypical M2 markers, were neither co-expressed nor correlated with higher or lower MHC class II expression levels in this study [34]. This further highlights the severe limitations of applying the in vitro M1/M2*-*concept to in vivo macrophages.

**Fig. 3:**
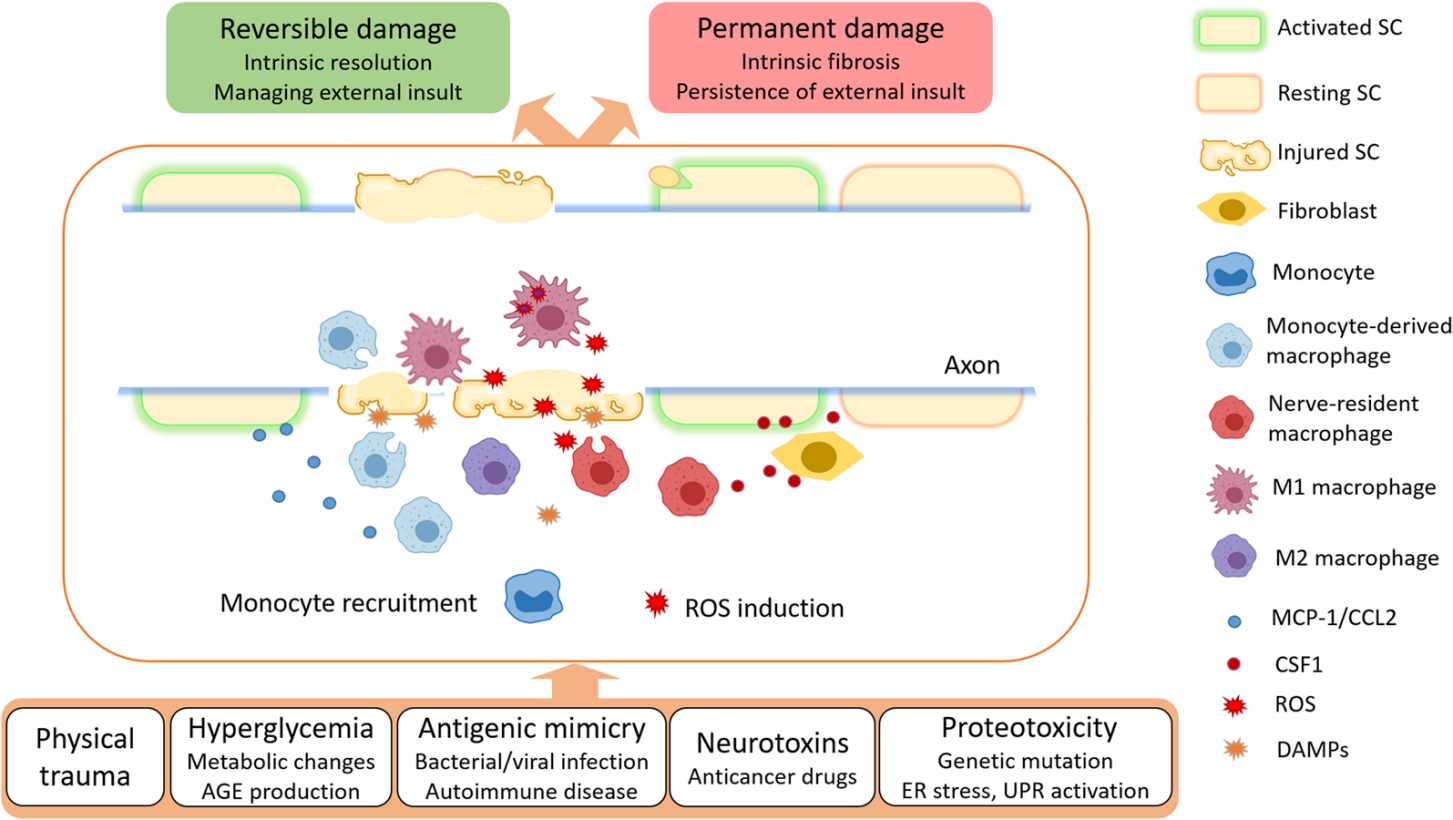
Schematic summary of the inflammatory process taking place in the peripheral axon. Monocyte recruitment and ROS production are common pathological mechanisms in many peripheral neuropathies of various causes. Several mechanisms take place simultaneously. Activated Schwann cells (SC) transdifferentiate to clear debris and recruit blood monocytes through the secretion of monocyte chemoattractant protein 1 (MCP-1/CCL2). In addition, repair SC and local fibroblasts express and secrete colony stimulating factor 1 (CSF1) to mobilize resident macrophages. Damage associated molecular patterns (DAMPs) also polarize macrophages. The on-site macrophages present a spectrum of phenotypes between M1-like macrophages and M2-like macrophages. Resolution of neuroinflammation (in a critical time-window) through intrinsic regulation or management of the external insult (e.g. correcting hyperglycaemia or withdrawing neurotoxins), likely results in reversible damage that only mildly affects nerve functionality. However, unresolved inflammation can cause tissue remodelling and fibrosis, severely affecting nerve function. AGE: advanced glycation end-products. ER: endoplasmic reticulum. UPR: unfolded protein response

Neurodegenerative diseases have a variety of causes but share some similar features: impaired mitochondrial function, increased oxidative stress and ROS production, inflammatory reactions, formation of protein aggregates and proteo-toxicity, and excito-toxicity. The counter-acting effects of axonal survival versus axonal degeneration factors determines whether an axon will be maintained or undergo self-destruction. In disease context, these events constitute a vicious cycle leading to neuron death [107]. In the following section, we discuss disease states in which inflammatory activity plays a direct causative role in peripheral neuropathies (PN), e.g. Guillain–Barré syndrome (GBS), as well as conditions where inflammation mediates PN secondary to underlying metabolic (diabetic polyneuropathy (DPN)) or genetic (Charcot–Marie–Tooth (CMT)) diseases or due to administration of anti-neoplastic drugs (chemotherapy-induced PN (CIPN)) (Fig. 3). As we will see, neuroinflammation is almost always accompanied by features of oxidative stress, which maintains or exacerbates the pathology. The interplay between macrophages and other cells in either development and continuation, or the correction of the disease milieu, will also be discussed, based on the available published data.

### Guillain–Barré syndrome and chronic inflammatory demyelinating polyradiculoneuropathy (CIDP)

A straightforward example of a disease state in which abnormal immune activation is the primary driver of pathological processes in peripheral nerves is Guillain-Barré syndrome (GBS). It is the most common cause of acute neuromuscular weakness and paralysis worldwide, and encompasses a group of acute immune-mediated disorders restricted to peripheral nerves and nerve roots. GBS is considered to be an autoimmune disease triggered by molecular mimicry following a bacterial or viral infection [108]. While the exact role of macrophages in GBS is still not fully understood, changes in BNB permeability, macrophage infiltration as well as macrophage-associated demyelination have all been described in GBS [109]. Auto-antibodies against SCs and axonal plasma membranes activate the complement cascade system that further recruits macrophages to the injury site [110]. Moreover, Th1 cells secrete TNFα which increases MCP-1 and intercellular adhesion molecule-1 (ICAM-1) expression, thus facilitating macrophage infiltration, recognition of SC and consequently myelin phagocytosis [111, 112]. Macrophage migration inhibitory factor (MIF) was also shown to play a critical role in the initiation and progression of GBS and its animal model [113]. More recently, invasion of the macrophage cytoplasmic processes into the internodes and nodal regions in sural nerves was observed in GBS patients. These sites were associated with macrophage-associated demyelination [114]. In the cerebrospinal fluid of human GBS patients, the median concentrations of inflammatory mediators, such as IL-8 and IL-1ra, as well as CCL2-7-9, CXCL9-10-12, and VEGF have been reported to be higher in comparison to healthy subjects [115]. Moreover, diminished lipophilic antioxidant defence, mainly γ-tocopherol and δ-tocopherol, in the plasma of GBS patients, leading to lowered resistance to ROS, is linked to the pathogenesis of GBS [116]. It is noteworthy that several cases of demyelinating GBS have been recently reported in patients with SARS-CoV-2 (COVID-19) through an autoimmune cross-reactivity mechanism [117]. Zika virus, also associated with the incidence of GBS, was also recently shown to trigger oxidative stress cellular responses in mice which could be a mediating factor in Zika virus infection and neurological complications [118]. While the recovery from GBS occurs over six months to two years [119], the chronic form of GBS presents progressive symptoms and results in chronic inflammatory demyelinating polyradiculoneuropathy (CIDP). As such, CIDP is characterized by a continuous cycle of self-propagating inflammation. Recent studies have underlined the importance of cell-mediated immunity in the pathological process, specifically T4 and T8 lymphocytes and macrophages, as well as the breakdown of the BNB and consequent autoantibodies and inflammatory cell infiltration [120]. Similar to GBS, demyelination caused by macrophages has been proposed to play an important role in the pathogenesis of CIDP [121]. Specifically, the presence of macrophage clusters around endoneurial blood vessels in sural nerves has been suggested as a diagnostic marker for CIDP patients compared to controls [122]. However, further studies are required in order to delineate the exact mechanisms of macrophage activation as well as their behaviour that culminate in the course of the disease process.

### Diabetic polyneuropathy (DPN)

It is well-established that hyperglycaemia activates inflammatory and oxidative stress pathways, thus creating the diabetic milieu. These two axes interact at several cell-signalling levels leading, in the long-term, to microvascular complications and peripheral nerve dysfunction, known as diabetic polyneuropathy (DPN) [123–125]. Uncontrolled persistent hyperglycaemia activates several metabolic pathways including polyol, protein kinase C (PKC), advanced glycation end-products (AGEs), and hexosamine pathways that alter the cellular metabolic state. AGEs stimulate microglia and macrophages to produce cytokines such as IL-1, IL-6, IL-17, TNFα, C reactive protein and chemokines such as CCL-2 and CXC [98]. In addition, hyperglycaemia-sensitive cells, such as endothelial cells, respond by increasing mitochondrial ROS and RNS production and vascular adhesion molecule formation. Furthermore, persistent hyperglycaemia modifies the glycosylation of myelin proteins, and thus their antigenicity, causing further infiltration and activation of monocytes, macrophages, and neutrophils, as well as ROS production. In contrast to the well-regulated ROS production in the anti-microbial response, metabolically generated ROS secretion in diabetic leukocytes is more impulsive and dysregulated. Macrophage infiltration further damages the myelin sheath and increases axon excitability, thus leading to oedema, inflammation, and cell necrosis. [98, 124, 125]. Under these conditions, Nrf-2 pathway has been shown to be suppressed leading to a downregulation in expression of antioxidant genes [124]. Therefore, persistent high glucose levels lead to a vicious circle of microvasculitis at nerve sites, neuroinflammation, and oxidative stress that damages peripheral nerves causing a broad range of sensori-motor symptoms.

### Chemotherapy-induced peripheral neuropathy (CIPN)

Chemotherapy-induced peripheral neuropathy (CIPN) is one of the most frequent secondary effects caused by anti-neoplastic drugs, with a prevalence ranging from 19% to over 85% [126]. CIPN occurs following treatments including vinca alkaloids (e.g. vincristine), platinum derivatives (e.g. cisplatin and oxaliplatin), and taxanes (e.g. paclitaxel). Importantly, pathological changes and symptoms vary with the dose, frequency, and physicochemical properties of the drug; and they are mainly sensorial, including pain, tingling, and numbness [127]. Although the mechanisms underlying CIPN are incompletely understood, inflammation- and oxidative stress-induced peripheral sensitization have been implicated as likely factors. CIPN is associated with mitochondrial alterations and increased intracellular ROS [128], as well as increased inflammatory cytokines [129] in dorsal root ganglia (DRG) sensory neurons. In rodent models, paclitaxel has been shown to trigger neuronal ROS production which consequently stimulates endogenous expression of the antioxidant enzyme superoxide dismutase-1 (SOD1) [130]. In addition, it is well-established, in both animal models and human patients, that chemotherapeutic agents stimulate involvement of the immune system in the anti-cancer effect [131]. The effect of non-neuronal cells, especially leukocytes, in CIPN is particularly important, given that in some cases only a weak correlation between CIPN and neuronal damage is observed, both in human patients and animal models [132]. Several studies on animal models of CIPN (notably paclitaxel-induced) indicate that symptoms are accompanied by activation of innate immunity signals, especially macrophages in the DRG and sciatic nerves [133–135], through activation of TLR4 signalling and increased expression of MCP-1 [135]. The influx of toxins and leukocytes through the BNB can be exacerbated as a consequence of breakdown of the BNB by matrix metalloproteinases (MMPs), some of which are upregulated by chemotherapeutic drugs. Administration of monoclonal anti-MMP9 antibodies can significantly decrease the expression of IL-6 and TNFα, two pro-inflammatory cytokines shown to be involved in paclitaxel-induced CIPN [136]. In a murine CIPN model, oxaliplatin was shown to stimulate the hypoxia signalling pathway through HIF-1, leading to MMP-9 activation and high expression of tissue factor (TF) and HSP70 in macrophages, thereby inducing a thrombosis-mediated circulatory disturbances in the sciatic nerve microenvironment [137]. More recently, oxaliplatin was shown to increase high-mobility group box-1 (HMGB-1) release from neurons and macrophages, which in turn induces MMP-9 release in neurons and macrophages, contributing to the progression of CIPN in mice [138]. Novel treatment strategies based on one or more target(s) in this network (i.e. microglia/macrophage inhibition, combating oxidative stress, blocking MMPs, and anticoagulants) achieved promising results in prevention/attenuation of CIPN symptoms in both animal and clinical studies [139].

### Charcot–Marie–Tooth disease

Charcot–Marie–Tooth (CMT) disease is a heterogeneous group of diseases constituting the most common hereditary pathology of the PNS. Genetic mutations are manifested as lesions in myelin (CMT1) or axons (CMT2), and symptoms are variable and include distal muscle weakness and sensory perturbations [140]. Neuroinflammation has been described in both demyelinating and axonal CMT. CMT1-related myelin gene mutations lead to SC cytotoxicity, impaired myelination and consequently to nerve pathology. Interestingly, studies in rodent models of CMT1X [141], CMT1A [142] and CMT1B [143] showed that mutant SC express MCP-1/CCL2 through the MEK-ERK signalling pathway that guides pathogenic macrophage infiltration. In addition, CSF-1 knockout rescued the phenotype of CMT1X mice by stabilizing macrophage invasion and therefore preserving SC homeostasis despite the mutation [144]. More surprisingly, important cell–cell contacts were observed between the CSF-1-expressing fibroblasts and endoneurial macrophages in the diseased nerve, posing more questions about the complex cellular and molecular interactions in peripheral nerves [145]. On the other hand, mutations in the mitochondrial GDAP1 (ganglioside-induced differentiation-associated protein 1) gene cause axonal CMT and are associated with increased production of ROS and inflammatory mediators [146]. Furthermore, oxidative stress has also been described in CMT1A patients [147] and a rat model [92] and is thus suspected to play a role in the pathological process. However, the link between oxidative stress and macrophages is as yet not established in CMT disease.

## Macrophages in therapy: potential and pitfalls

We have presented here the different phenotypes of macrophages depending on their microenvironment. These phenotypes have differential effects on the cellular biology and disease progression. The divergence in such phenotypes provides a therapeutic potential of monocyte/macrophage manipulation for the treatment of several pathologies including cancer, metabolic, autoimmune and neuroinflammatory diseases, as well as for regenerative medicine. This approach is already the subject of extensive study. For our purposes, macrophages are particularly relevant in therapeutics as these cells have (i) inherent plasticity that is well-suited to therapeutic manipulation; (ii) phagocytic abilities allowing them to be efficiently targeted with nanoformulated compounds, and (iii) critical roles in tissue repair. Among the possible therapeutic strategies, cell therapy by ex vivo-activated macrophages, or delivery of molecules and biomaterials to modulate the accumulation and phenotype of endogenous macrophages have been developed [148]. Attempts to inhibit the pro-inflammatory M1-like phenotype or promote an anti-inflammatory M2-like phenotype, by modulating the activity of inflammatory cytokines and transcription factors, have also been investigated. In diabetic mice, anti-IL-1β antibodies were shown to downregulate the M1-like phenotype and promote the M2-like phenotype, thus improving wound healing [149]. Similarly, topical application of peroxisome proliferator-activated receptor (PPAR)-γ agonists, known to promote an M2-like phenotype [89], was shown to improve wound healing in diabetic mice [150]. In a similar fashion, systemic M-CSF administration has been reported to promote remyelination following spinal cord injury in mice [151]. Likewise, transplantation of dental pulp stem cells suppressed inflammation in rat sciatic nerves by promoting macrophage polarization towards an anti-inflammatory phenotype and ameliorated diabetic polyneuropathy [152].

In a clinical context, data from the PNS are still lacking. Nevertheless, some clinical data have been collected from trials in different organ systems. For example, niacin administration in patients with Parkinson’s disease was accompanied by improved quality of life through the attenuation of inflammation by shifting macrophage polarization from an M1 to M2 profile [153]. Moreover, the delivery of ex vivo-activated autologous M2 macrophages was shown to be beneficial in patients with heart failure [154] or stroke [155]. Multiple experimental and clinical studies have shown a protective role of MIF in GBS [113], although clinical trials are still unavailable. Several clinical trials have used parameters related to the macrophages, such as cytokine secretion, as primary or secondary clinical outcomes or biomarkers (for example *NCT03321955*).

It should be noted, however, that monocytes/macrophages may be less responsive to stimuli in the elderly and in patients with immune disorders, suggesting that macrophage-recruiting strategies may be less effective in these subjects. While targeting/using macrophages for therapy in diabetes, heart and CNS diseases shows great potential, macrophage manipulation in the context of the PNS should be seriously considered and its investigation encouraged for the development of next generation therapeutics. However this will also requires more efforts in the screening for biological effects using a combination of in vitro and in vivo assays.

## Conclusions

It has now been fully accepted that monocytes are not direct precursors for many tissue-resident macrophages. Indeed, monocyte provision during homeostasis contributes partially to the tissue-resident macrophage pool, part of which is seeded prenatally [18]. However, the tissue-specific local environment is now known to be the most powerful controller of macrophage phenotype, regardless of their origin. Several models have been suggested to explain macrophage differentiation, including their day-to-day function, maintenance, population density and interaction with the surrounding microenvironment (or niche) [1]. These debates reflect the level of complexity and heterogeneity of these immune cells in tissue. For example, within a single tissue, macrophage heterogeneity is potentially underestimated due, in part, to the challenges involved in investigating small cell subsets, and the possible contamination of samples by other cells. New sophisticated techniques of single-cell sequencing and fate mapping, are now helping to overcome these limitations and are providing a significant contribution to the field in the so-called “single-cell era” [156]. Although limited, thus far, in comparison to their more extensive use in the study of CNS cells, applying these approaches to the PNS will certainly provide valuable information about the spatial, temporal, and functional distribution of tissue macrophages. These characteristics are expected to be even more complex in the pathological context. As data are lacking regarding this point, macrophage recruitment and origin in pathological states is an interesting substance for future investigation. Moreover, the current view that macrophages interact with primary sensory neurons in the peripheral tissues and the DRG to regulate not only inflammatory responses but also pain signals requires further study. One should keep in mind the difficulties in preparing libraries and analysing the large amount of data generated by single-cell sequencing [157]. Collaborative approaches across the globe such as the Human Cell Atlas [https://www.humancellatlas.org] are creating an extensive catalogue to describe every single human cell type. Another challenge in studying macrophages associated with the PNS is the wide distribution of peripheral nerves within various tissue compartments; hence nerve-associated macrophages are exposed to distinct environmental signals. Indeed, epigenetic imprinting cannot be captured by transcriptomic or proteomic techniques, necessitating the synergistic use of other techniques to assess chromatin state. Recent compelling studies, in germ-free mice and/or adult mice treated with antibiotics, have emphasized the role of the microbiome in the time-dependent control of CNS microglia maturation and function [158–160]. Hence, what might be the corresponding role of host microbiota in regulating PNS macrophage phenotype? This question requires future investigation.

On the other hand, the regeneration potential of the mammalian nervous system has been the subject of extensive research. While the acute inflammatory phase is destructive to peripheral nerves, proper WD, through immune cell clearance of myelin and axonal debris, is crucial for the repair process. Following insults to peripheral nerves, monocytes are recruited to the site of injury and resident macrophages are activated. These processes collectively termed neuroinflammation can be ultimately beneficial or detrimental to nerve function. Despite the regenerative capacity of PNS axons, clinical experience attests to pathological tissue remodelling and to disappointing functional recovery. The complex relationship between SCs, the axon, macrophages and endoneurial fibroblasts is the driver of these outcomes. In comparison to the CNS, nerve regeneration in the PNS may be incorrectly thought to be complete with little intervention [9]. However, successful PNS regeneration depends on the age of the patient and the delay before intervention, as well as on local factors, essentially the type, site, and severity of the injury, the recruitment of non-neuronal cells (fibroblasts and inflammatory cells), and the local microenvironment [7, 9]. Learning how to harness the benefits of neuroinflammation by polarizing macrophages into their regulatory/anti-inflammatory phenotype or enhancing the elimination of debris is currently the subject of extensive research. Moreover, we should stress that the interaction of the four local cell types mentioned above seems to be crucial in both physiological and pathological situations, making these interactions a good, yet challenging, target for potential therapeutics. In the future, directing research towards the modulation of macrophage phenotype may identify ways to help alleviate and reverse peripheral nerve insults through targeting both neuroinflammatory and oxidative stress pathways.

## Data Availability

Not applicable.

## Abbreviations

BNB: Blood–nerve barrier
CIPN: Chemotherapy-induced peripheral neuropathy
CMT: Charcot–Marie–Tooth disease
CNS: Central nervous system
CSF1: Colony stimulating factor-1
DPN: Diabetic polyneuropathy
DRG: Dorsal root ganglia
ECM: Extracellular matrix
GBS: Guillain–Barré syndrome
HSC: Hematopoietic stem cells
IL: Interleukin
MCP-1: Monocyte chemoattractant protein 1
NGF: Nerve growth factor
NO: Nitric oxide
PN: Peripheral neuropathies
PNS: Peripheral nervous system
ROS: Reactive oxygen species
SC: Schwann cell
TNFα: Tumour necrosis factor-α
VEGF: Vascular endothelial growth factor
WD: Wallerian degeneration
